# Key aspects related to implementation of risk stratification in health care systems-the ASSEHS study

**DOI:** 10.1186/s12913-017-2275-3

**Published:** 2017-05-05

**Authors:** Joana Mora, Miren David Iturralde, Lucía Prieto, Cristina Domingo, Marie-Pierre Gagnon, Catalina Martínez-Carazo, Anna Giné March, Daniele De Massari, Tino Martí, Marco Nalin, Francesca Avolio, Jean Bousquet, Esteban de Manuel Keenoy, all ASSEHS group, E. de Manuel, J. Mora, M. David, L. Prieto, A. Fullaondo, I. Erreguerena, C. Martinez-Carazo, A. Giné March, C. Domingo, E. Millan, J. Orueta, M. Ogueta, S. Esnaola, E. Valıa, F. Rodenas, S. Pauws, J. op den Buijs, D. De Massari, M. Asim, J. Contel, T. Martı, I. Baroni, M. Romano, M. Nalin, F. Avolio, F. Robusto, V. Lepore, J. Bousquet, A.. Bedbrook, R. Bourret

**Affiliations:** 1 Kronikgune-Centro de Investigación en Cronicidad, Bilbao, Basque Country Spain; 2 Osakidetza-Basque Health Service, Bilbao, Spain; 3 Laval University, Québec, Canada; 4 Philips Electronics Nederland B. V., Eindhoven, Netherlands; 5 Fundació Ticsalut, Catalonia, Spain; 6 Telbios S.p.a., Milan, Italy; 7 ARES Puglia, Gallipoli, Italy; 8 Centre hospitalier régional universitaire de Montpellier, Montpellier, France; 9 Kronikgune -Centro de Investigación en Cronicidad, Torre del BEC, Ronda de Azkue, 1, 48902 Barakaldo, Bizkaia Spain

## Abstract

**Background:**

The lack of proven efficacy of new healthcare interventions represents a problem for health systems globally. It is partly related to suboptimal implementation processes, leading to poor adoption of new interventions. *Activation of Stratification Strategies and Results of the interventions on frail patients of Healthcare Services* (ASSEHS) EU project (N° 2013 12 04) aims to study current existing health Risk Stratification (RS) strategies and tools on frail elderly patients. This paper aims at identifying variables that make the implementation of population RS tools feasible in different healthcare services.

**Methods:**

Two different methods have been used to identify the key elements in stratification implementation; i) a Scoping Review, in order to search and gather scientific evidence and ii) Semi-structured interviews with six key experts that had been actively involved in the design and/or implementation of RS strategies. It aims to focus the implementation construct on real-life contextual understandings, multi-level perspectives, and cultural influences.

**Results:**

A Feasibility Framework has been drawn. Two dimensions impact the feasibility of RS: (i) Planning, deployment and change management and (ii) Care intervention. The former comprises communication, training and mutual learning, multidisciplinarity of the team, clinicians’ engagement, operational plan and ICT display and functionalities. The latter includes case finding and selection of the target population, pathway definition and quality improvement process.

**Conclusions:**

The Feasibility Framework provides a list of key elements that should be considered for an effective implementation of population risk stratification interventions. It helps to identify, plan and consider relevant elements to ensure a proper RS implementation.

**Electronic supplementary material:**

The online version of this article (doi:10.1186/s12913-017-2275-3) contains supplementary material, which is available to authorized users.

## Background

European Health Systems and services move towards proactive, anticipatory and integrated care [1, 2]. Health care systems need to provide services using an adequate level of resources. Population health management is enhanced by using tools to stratify people with chronic diseases and/or frailty according to their risk [2–6]. Risk Stratification (RS) has a long history in American health systems. [7] Its use in European public health care provision environments is still at an initial stage. There is a growing number of research work and scientific literature about RS methodologies in Europe. Most of them relate to the validation of risk algorithms. [4, 8] There are few descriptions of the implementation process [8] or policy experiences [9]. The identification of the key issues in RS implementation can help the organizations to optimize the process.

RS tools can help to identify complex high-risk patients and maintain these patients on the radar of the Health Services and enhance Population health management [10]. It facilitates appropriate coverage of proactive health interventions. It also boosts the coordination between primary, secondary and social care. RS can identify patients that can benefit most of common shared objectives between the different providers.

Multi-morbid patients use up to 50 times more health care resources than non-chronic patients [11]. This is partly due to the reactive and fragmented way in which care is delivered [12]. Multidimensional and multidisciplinary integrated care approaches are more effective and efficient to ensure quality and continuity of care [12]. They can diminish or delay the occurrence of unwanted events and improve patient’s wellbeing and system sustainability [13].

The suboptimal implementation processes of new interventions, leads to their diminished efficacy. Implementation research has emerged the last decade to help understand the nature of these problems and narrow down the gap between knowledge and practice [14]. Implementation of evidence-based interventions is not always achieved [15]. Feasibility has been identified, among others, as a key area in the design and implementation of evidence-based interventions [16].

Activation of Stratification Strategies and Results of the interventions on frail patients of Healthcare Services (ASSEHS) EU project (N° 2013 12 04) [17] is an international attempt to bring together professionals involved in risk stratification work from Health Services, Academia and Research centres of European Countries. The aims were to study current health risk stratification strategies and tools and to understand the challenges involved in extending their use on frail elderly patients. ASSEHS is in line with the B3 Action Plan of the European Innovation Partnership on Active and Healthy Ageing (EIP on AHA).

### Objectives

General objective: to draw a framework to assess the feasibility of implementing Population Risk Stratification strategies.

Specific objectives:

- ▪ To identify the key elements described in the literature, focusing on barriers and facilitators at the macro, meso and micro levels of management and clinical practice in healthcare systems.
- ▪ To structure the key aspects identified within a framework relevant to the feasibility of risk stratification implementation.

## Methods

Two different methods have been used to identify the key elements in stratification implementation; i) A Scoping Review, in order to search and gather scientific evidence and ii) Semi-structured interviews with six key experts that had been actively involved in the design and/or implementation of RS strategies.

### Scoping review

A scoping review has been carried out following a five step procedure [18]. It comprised: 1) Identifying the research question, 2) Identifying relevant studies, 3) Selecting the key studies, 4) Charting the data, 5) Collating, summarizing, and reporting result. For further information of the scoping review, see Mora J et al. [19].

Only documents describing RS implementation experiences or addressing key aspects of this process were eligible. The scoping review was conducted in May 2014. Searches were carried out by an expert medical librarian.

#### Identifying the research question

The first step was to identify and assess the key terms of search in natural language (See Additional file 1: Annex 1_ Search question). Then, a strategy of generic search was defined, composed by controlled vocabulary (Medical Subject Headings designed-MeSH, Emtree and other thesauri terms) and free speech, considering synonyms, abbreviations, acronyms, and plural spelling variations, later finding was adjusted redefining and adjusting to the most relevant terms.

The strategies were complemented by field identifiers, wildcards, proximity operators and Boolean operators. This strategy was validated through a virtual consultation with experts and was adapted later to the different sources of information and selected resources. (See Additional file 2: Annex 2: Search Strategy).

The search strategy was tested in a single database (Pubmed) to ensure that the terms and connectors chosen provided relevant results for the scope of the research. No further changes were introduced in the search strategy. It was then applied in other relevant databases:

- MEDLINE (Pubmed)
- EMBASE.com
- The Cochrane Library (Wiley platform)
- CINAHL
- PsycINFO
- CRD
- Google scholar
- TripDatabase
- Lilacs

To facilitate reading and data analysis, language of the publications was restricted to Spanish, English and French.

#### Identifying relevant studies

The implementation of the search strategy in the selected databases resulted in 982 papers found. In a first screening, the title and abstract were analyzed to ensure their eligibility within the RS subject. Two hundred one papers were selected for further analysis.

#### Selecting key studies

A second screening was done, analyzing if the papers were focused on RS implementation. The analysis was based on the title and abstract, and reading of the complete paper when needed. Seventy three papers were prioritized. They included implementation information such as:

1. Can we identify interventions (in Europe) where RS approaches have been used?
2. Do they describe the implementation process?
3. Do they provide information on barriers and facilitators?
4. Which are the barriers?
5. Which are the facilitators?
6. How to overcome barriers?
7. Where else can we find this information?
8. Can we identify names of Key Informants?

Forty three papers were added to the search through a snowball process. Snowballing refers to using the reference list of a paper or the citations to the paper to identify additional papers [20].

All 116 papers were read (73 plus 43). Thirty four papers relevant to the process of implementing a RS tool in real life were finally selected.

**Fig. 1:**
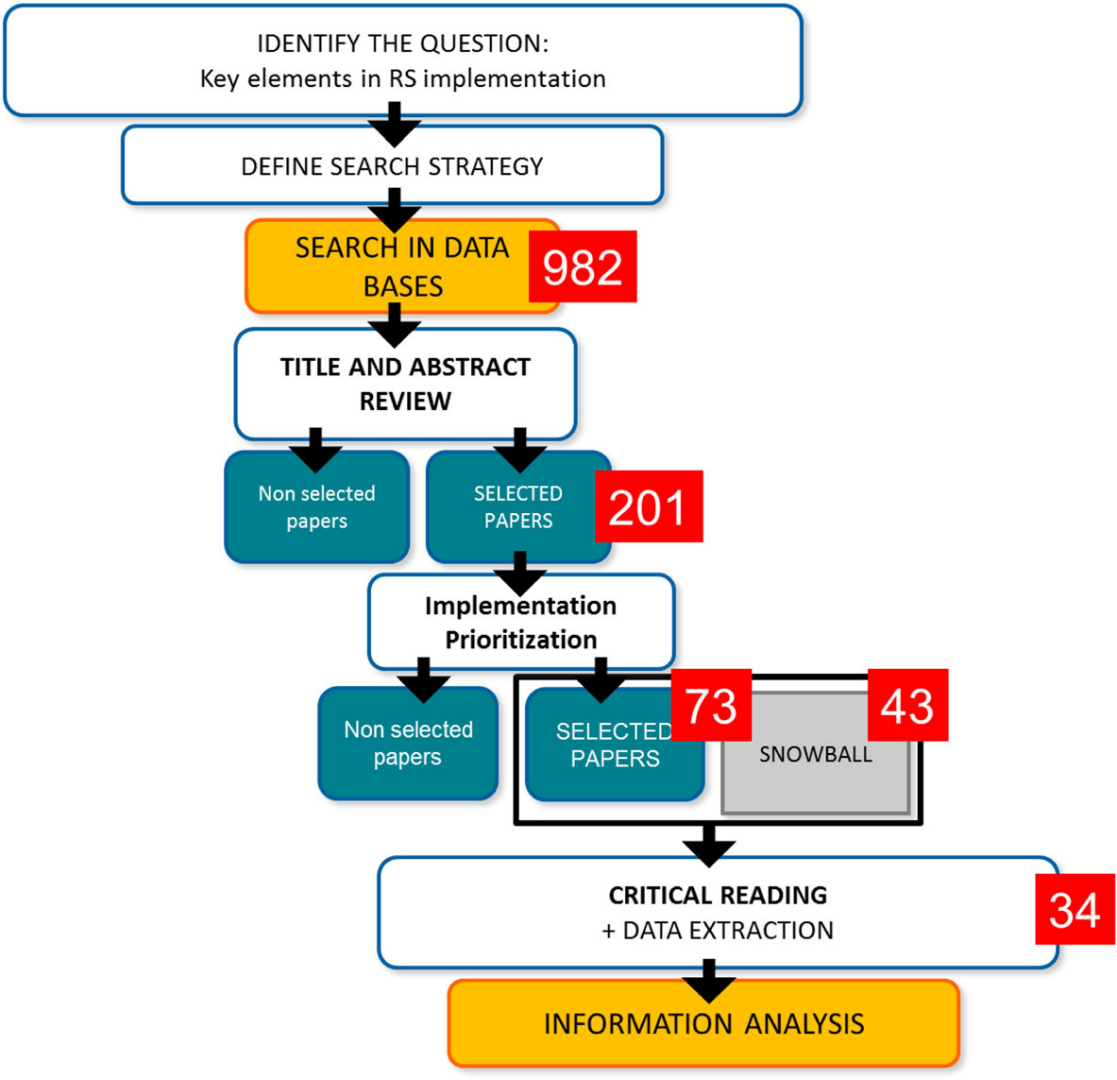
Scoping Review Process Workflow

Figure 1 shows a diagram of the process followed for the selection of the papers for critical reading. The screening of the papers at all stages was made by peers (JM, MD, LP, CD), and disagreements were solved through further discussion and analysis between the individuals or through the view of a third one.

#### Charting the data: table of evidence

A table of evidence was designed to identify elements relevant in the process of RS implementation. Critical reading of the 34 papers selected was performed by four peers. Each paper was read at least by two people. The table of evidence contained general information of the source (name of the document or type of information described,) and the key implementation aspects identified.

#### Collating, summarizing and reporting results

A thematic analytic construction was used to identify prominent or recurrent topics, summarize the findings and develop categories under thematic headings. Summary tables, providing descriptions of the key points were produced [21]. Items related with implementation were identified in each paper. They were grouped in broader topics. Information related to the features and impact of the tool was excluded. A comparison-iterative method of content analysis was performed. Each category was searched for in the entire data set and all instances were compared, until no new categories could be identified [22]. Categories were grouped according to their subject. They were integrated in two major dimensions, one comprising planning and organization categories, the other those more related with clinical management.

### Interviews with key experts

To further develop and validate the scoping review findings, a qualitative methodology approach was used. It aimed to understand concepts in their natural context, emphasizing the meaning, experience and views of experts in this area. The purpose is to focus and the implementation construct on real-life contextual understandings, multi-level perspectives, and cultural influences [23].

Semi-structured interviews with six experts on implementation of population risk stratification strategies in health care systems were done [24]. They explored the conditions of a system that allow optimal introduction of risk stratification tools in the Health System, the facilitators and barriers in the process and to identify mistakes made in implementation or that could affect the implementation.

Qualitative data analysis included literal transcription and content analysis of narratives by inductive method of reading and re-coding [25]. With these results the dimensions and categories of the framework were reexamined, as a check of validity, focusing in identifying new relevant items for inclusion or changes in the meaning and boundaries of the previous categories [26]. The dimensions were defined according to the phase of the implementation process. Categories were allocated to each of these phases.

## Results

### Results of the scoping review

Thirty four articles were selected on the scoping review (Fig. 1). In the process of critical reading, different recurrent items were identified and summarized in 34 topics (Table 1). These topics were further synthesized according to their subject through a comparison-iterative process, identifying similarities or differences, into a first framework draft with 23 categories. Each category could comprise one or more topics, depending on their meaning, scope and association. A new iteration allowed to connect the categories in broader headings and culminated in a framework with seven dimensions (Table 2).

**Table 1:** Topics identified

Type of study	Information support
Data sources	Key players (teamwork, leadership)
Data access	Timing
Methodological support	Outcomes
ICT visualisation	Change management
Training/mutual learning	Payment per outcomes
Quality Assessment- Evaluation - follow up	Patient enrolment/recruitment
Risk prediction outcomes	Patient follow-up
Deployment strategy	Impactable patients
Target population	Incentives
Refinement/validation of the RS tool	Workload
Knowledge of patients portfolio	Clinicians involvement
Planning	RS functionalities
Communication	Ethical issues/Conflict of interests/equity
RS update	Patient activation/engagement
Patient selection and identification	Budget distribution
Cost/Financing	Intervention

**Table 2:** Draft framework

Dimensions	Categories	Topic	Papers
Cost	Cost	Cost/Financing	[5, 32–41]
	Timing	Timing	[38]
Ethics	Ethical issues	Ethical issues/Conflict of interests/equity	[32, 34, 37, 39, 42–46]
Funding and resource allocation	Funding and resource allocation	Budget distribution	[39, 44, 45]
		Resource redistribution	[32, 34, 35, 38–40, 44, 45]
	Revisions of the reimbursement model	Payment per outcomes	[41, 44, 45]
Key aspects of care intervention	Case finding	Impactable patients	[37, 42–44, 47–53]
		Patient activation/engagement	[34, 54]
		Patient enrollment/recruitment	[5, 40, 44, 50, 51, 55]
		Patient selection and identification	[5, 32, 37, 38, 41–44, 46–51, 56, 57]
	Case finding/data accuracy	Target population	[5, 34, 38, 41, 45, 51, 54]
	Case finding/funding and resource allocation	RS functionalities	[5, 32, 36, 37, 39, 43, 46, 47]
	Pathway definition and implementation	Intervention	[35, 46]
		Patient follow-up	[47, 50]
Others	Others	Knowledge of patients portfolio	[5, 35, 36, 38, 42, 44, 51]
Planning, deployment and change management	Clinicians engagement	Change management	[32, 34, 37, 40–43, 47, 51]
		Clinicians involvement	[5, 34, 38, 42, 54]
		Incentives	[32, 33, 37, 41, 58]
		Methodological support	[38, 45]
		Workload	[5, 54]
	Communication	Communication	[5, 34, 38, 51, 58]
	ICT visualization	ICT visualization	[5, 35, 38, 40, 41, 43, 47, 50, 51, 57]
		Information support	[35, 38, 47, 50, 51]
	Multidisciplinary team for RS deployment	Key players (teamwork, leadership)	[5, 35, 38, 43]
	Quality assessment and improvement process	Quality Assessment- Evaluation - follow up	[32, 33, 38, 41, 43, 47]
	Operational plan	Deployment strategy	[5, 35, 38]
		Planning	[5, 32, 34, 37, 38, 40, 41, 46]
	Training and mutual learning	Training/mutual learning	[5, 33, 35, 36, 38, 43, 50, 51]
RS information	Data accuracy	Refinement/validation of the RS tool	[5, 34, 38, 40, 46]
	Data availability	Data access	[5, 36, 38, 40, 47, 51, 53, 56, 58]
	Data source	Data sources	[5, 32–43, 46–49, 51, 53–59]
	Outcomes	Health related outcomes	[33, 36, 38, 41, 51, 57, 58]
	Risk tool outputs	Risk prediction scores	[5, 32–34, 36, 38, 39, 41, 43, 44, 46, 47, 51, 56]
	Updating frequency	RS update	[5, 33, 38, 42–46, 49, 51]

This first framework draft includes many elements of an intervention implementation such as costs, ethics, planning, deployment and change management.

### Results of the semi-structured interviews to key experts: refinement of the assessment framework

Practical information generated through the implementation process of RS strategies was collected in order to refine the framework developed during the scoping review. The relevant concepts related to facilitators and barriers relevant in implementation were identified. Meanings and categories’ boundaries were redefined. The dimensions were defined according to the focus in the implementation process, planning and organization for one side and clinical management for the other. Categories were allocated to each of these dimensions.

### Final feasibility framework

The final framework was reduced to two dimensions (Table 3). The first one includes all the categories related with planning, organizational and managerial issues. The second one comprises those aspects related to patient selection and clinical care.

**Table 3:** Feasibility Final Framework

Dimensions	Categories	Papers
Planning, deployment and change management	Communication	[5, 34, 38, 51, 58]
	Training and mutual learning	[5, 33, 35, 36, 38, 43, 50, 51]
	Multidisciplinarity of the team leading RS deployment	[5, 35, 38, 43]
	Clinicians’ engagement	[5, 32–34, 37, 38, 40–43, 45, 47, 51, 54, 58]
	Operational plan	[5, 32, 34, 35, 37, 38, 40, 46]
	ICT - Information display and functionalities	[5, 35, 38, 40, 41, 43, 47, 50, 51, 57]
Care intervention	Case finding/Selection of the target population	[5, 32, 34, 37, 38, 40–44, 46–52, 54–57]
	Pathway definition and implementation	[35, 46, 47, 50]
	Quality assessment and improvement process	[32, 33, 38, 41, 43, 47]

Planning, deployment and change management dimension includes six categories. Communication involves the process of explaining the purpose and outcomes of RS to health professionals. Training and mutual learning comprises the activities performed so as the professional become competent in the use of RS. Multidisciplinarity has to do with the degree the team leading RS deployment. It includes a variety of health professionals, managers, ICT professionals, epidemiologists and others. Clinicians’ engagement refers to topics such as the mechanisms and degrees of professionals’ accountability, commitment and involvement. Operational plan focuses on the way resources, activities, quality and implementation has been defined. ICT-Information display and functionalities includes the devices and applications used, their purpose, usability, flexibility, performances and support.

Care intervention includes three categories. Case finding is the selection, identification and enrollment of the target population. The pathway definition and implementation involves the organized clinical intervention processes with the patients including the follow up and monitoring. Quality assessment and improvement process is related to the evaluation and changes introduced during the implementation process.

## Discussion

The poor implementation of interventions of proven efficacy is an issue for health systems. Implementation research tries to understand these problems and close the gap between knowledge and practice [27]. It is defined as the scientific study of strategies aimed at promoting the adoption of clinical research findings in routine clinical practice in a systematic, widespread, sustainable and continued way [28]. Identifying the key aspects related to implementation will help to better deploy risk stratification tools in health care systems [29]. ASSEHS has developed a framework using a scoping review together with an experts’ consultation process. They are complementary methods that contribute to a comprehensive approach to the problem.

The Feasibility Framework refinement was based on experts’ information. It is information rooted in real life circumstances. The Feasibility Framework elements should be considered for an effective implementation of population risk stratification interventions. They can be applied to in any health systems.

Planning, deployment and change management is one of those dimensions. A high-quality operational plan establishing the agenda and the strategic goals and objectives is needed. Having trained people qualified in RS is “a must” [30]. Clinicians’ engagement is a sine qua non requirement. If we can achieve the engagement of innovators and early adopters, the rest will follow their steps. Communication, not only of the RS tool’s characteristics, but also of its aims, is a key element for its feasibility [5]. Clinicians have to see the point of RS. Otherwise it will be really difficult to implement. Clinical group consists of different profiles. It is indispensable to have a multidisciplinary team leading the RS deployment [1]. Each and every one of the professional profiles involved is important. Good ICT systems have been identified as critical in risk stratification deployment.

Care intervention dimension has a paramount importance. Case finding and selecting the population groups allows focusing efforts and resources. The aim is to target persons that can get more benefit from programs designed for chronic patients [31]. Pathway definition and implementation have to be considered. Continuous improvement procedures, including quality assessment and improvement processes, enhances feasible RS interventions.

These findings are in line with the Consolidated Framework for Implementation Research [13]. It establishes a list of constructs that have been associated with effective implementation of evidence based interventions.

There are limitations in this study. Implementation of risk stratification tools involves other aspects apart from feasibility. Aspects related to the quality and availability of the data or the stratification algorithms has not been dealt with. Another limitation is that the number of experts did not ensure information obtained reached the point of data saturation. Experts’ empirical experiences were focused on their concrete reality. To reduce these biases, efforts were made to include worldwide references and publications during the literature review. The study has been based on NHS tyoe system. The experience of RS in insurance based health systems, has to be further studied.

## Conclusions

RS implementations feasibility is related to two different areas: organizational and management factors and patient selection and clinical care. The implementation strategy has to include planning of resources and organization of the deployment. Different issues regarding clinicians are critical and should not be underestimated. The functionalities of the ICT tools and the quality management of the process should not be lost of sight.

RS for health care provision has a long history in private health systems, but its application in public health care provision environments is still in an initial stage. Identifying the elements to consider in the implementation of RS can help to optimize its deployment and adoption. This framework is a conceptual model offering a broader theoretical understanding of risk stratification implementation. It aims to help to plan and guide the process of its deployment.

## Additional files

Additional file 1: Annex 1. Search question into PICO format. Search question organized in Problem/target population, Tool/Intervention, Comnparator and Outcomes. (DOCX 14 kb)

Additional file 2: Annex 2. Search Strategy. Key words used in the literature scoping review. (DOCX 13 kb)
