# Supplementary material for: Key aspects related to implementation of risk stratification in health care systems-the ASSEHS study
Source: BMC Health Serv Res. 2017 May 5;17:331. doi: 10.1186/s12913-017-2275-3 (PMC5420130; doi:10.1186/s12913-017-2275-3)
Supplement: Supplementary file 1 — Search question into PICO format. Search question organized in Problem/target population, Tool/Intervention, Comnparator and Outcomes. (DOCX 14 kb) [file 12913_2017_2275_MOESM1_ESM.docx]

**Annex 1: Search question into PICO format**

| **Target problem/population** | **Tool/intervention** | **Comparator** | **Outcomes** |
| --- | --- | --- | --- |
| Chronic conditions  Chronic disease  Chronic patients  Comorbidity  Elderly  Frailty  Functional decline  Multimorbidity  Old people  Polypathology  population  Primary care  Secondary Care  Specialized care | Adjusted Clinical groups  ACG  Adjusted risk model  Algorithm  Case-mix system  Combined predictive model  Continuity of care  Information  Integrated Care  Interventions  management  Model  Modelling  Patient classification  Risk adjustment  Risk prediction  Risk profiling  Risk stratification  Risk stratification model  Risk tool  Screening tool  Tailored intervention  Targeted intervention  Targeting | Not applicable to ASSHES | Implementation  Feasible  Feasibility  Barrier  Facilitator  Problem  Solution  Tackle  Challenge  Introduction  Scale up  Clinical acceptance  Understanding  Comprehensive  Routine practice |
