# Supplementary material for: Key aspects related to implementation of risk stratification in health care systems-the ASSEHS study
Source: BMC Health Serv Res. 2017 May 5;17:331. doi: 10.1186/s12913-017-2275-3 (PMC5420130; doi:10.1186/s12913-017-2275-3)
Supplement: Supplementary file 2 — Search Strategy. Key words used in the literature scoping review. (DOCX 13 kb) [file 12913_2017_2275_MOESM2_ESM.docx]

**Annex 2: Search Strategy**

("Chronic Disease"[Mesh] OR "Comorbidity"[Mesh] OR "Frail Elderly"[Mesh] OR “Frailty”[Title/Abstract] OR “Functional Decline”[Title/Abstract] OR “Multimorbidity”[Title/Abstract] OR “polypathology”[Title/Abstract] OR “high risk patient”[Title/Abstract] OR "Population at Risk"[ Title/Abstract])

AND (“Risk Adjustment"[Mesh] OR “Risk prediction”[Title/Abstract] OR “Risk profiling”[Title/Abstract] OR “Risk Screening”[Title/Abstract] OR "Patient Identification Systems"[Mesh] OR “Patient identification”[Title/Abstract] OR “Predictive modelling”[Title/Abstract] OR “risk stratification”[Title/Abstract] OR "stratify* risk"[Title/Abstract] OR “risk stratify*”[Title/Abstract] OR "Risk Assessment/methods"[Mesh] OR "Risk Assessment/standards"[Mesh] OR "administrative database"[Title/Abstract] OR "health care record"[Title/Abstract] OR "Medical Records Systems, Computerized"[Mesh] OR "Medical Records"[Mesh:NoExp]OR "Registries"[Mesh] OR registry[Title/Abstract])

AND

("Health Plan Implementation"[Mesh] OR "Regional Health Planning"[Mesh] OR “Implementation”[Title/Abstract] OR “Feasible”[Title/Abstract] OR "Feasibility Studies"[Mesh] OR “Feasibility”[Title/Abstract] OR “Barrier”[Title/Abstract] OR “Facilitator”[Title/Abstract] OR Tackle[Title/Abstract] OR "Scale up"[Title/Abstract] OR "Clinical acceptance"[Title/Abstract] OR "Routine practice"[Title/Abstract] OR “Deployment"[Title/Abstract] OR "Communication Strategy”[Title/Abstract] OR "Uptake"[Title/Abstract] OR Adoption[Title/Abstract])

NOT ("Animals"[Mesh] NOT ("Animals"[Mesh] AND "Humans"[Mesh]))
